# Unpacking KDIGO Guidelines: Prioritizing and Applying Exposures and Susceptibilities for AKI in Clinical Practice

**DOI:** 10.3390/jcm14082572

**Published:** 2025-04-09

**Authors:** Vicky Jenny Rebecka Wetterstrand, Thomas Kallemose, Jesper Juul Larsen, Lennart Jan Friis-Hansen, Lisbet Brandi

**Affiliations:** 1 Department of Endocrinology and Nephrology, North Zealand University Hospital, 3400 Hillerød, Denmark; lisbet.brandi@regionh.dk; 2 Department of Clinical Research, Copenhagen University Hospital Amager and Hvidovre, 2650 Hvidovre, Denmark; thomas.kallemose@regionh.dk; 3 Department of Emergency Medicine, North Zealand University Hospital, 3400 Hillerød, Denmark; jesper.juul.larsen@regionh.dk; 4 Department of Clinical Microbiology, Rigshospitalet, Copenhagen University Hospital, Institute of Clinical Medicine, University of Copenhagen, 1172 København, Denmark; lennart.jan.friis-hansen@regionh.dk

**Keywords:** AKI, NGAL, KDIGO, CRP, emergency department, AKI susceptibilities, AKI exposures

## Abstract

**Background/Objectives:** Acute kidney injury (AKI) is a significant global health issue with a high morbidity and mortality. The Kidney Disease: Improving Global Outcomes (KDIGO) guidelines identify various exposures and susceptibilities as risk factors for AKI. However, the predictive significance of these factors in heterogeneous emergency department (ED) populations remains unclear. We hypothesized that assessing KDIGO-listed exposures and susceptibilities for AKI, alone and in combination, would provide an insight into their predictive value for AKI. Furthermore, we investigated whether adding biomarkers, plasma neutrophil gelatinase-associated lipocalin (pNGAL) and C-reactive protein (CRP), could enhance AKI risk prediction. **Methods:** Data were analyzed from the prospective longitudinal “NGAL study” conducted at North Zealand University Hospital in Denmark. A total of 344 ED patients were included, with AKI diagnosed using KDIGO’s creatinine-based criteria. Patient data, including medical history, exposures, and susceptibilities, were extracted and analyzed. Predictive performance was evaluated using a receiver operating characteristic (ROC) analysis on individual and combined risk factors. Additional models incorporated pNGAL and CRP to assess their impact on prediction accuracy. **Results:** Individual exposures and susceptibilities showed a poor predictive performance, with nephrotoxic drugs and advanced age demonstrating the highest sensitivity but a low positive predictive value (PPV). Combining multiple risk factors improved AKI prediction, with models clustering into those optimizing sensitivity or PPV. The inclusion of pNGAL significantly enhanced predictive performance, achieving the highest combined sensitivity and PPV. Although less than pNGAL, CRP also improved prediction, while requiring fewer variables than pNGAL-inclusive models. **Conclusions:** No individual KDIGO-listed exposure or susceptibility could reliably predict AKI in the ED setting. Combining multiple exposures and susceptibilities improved the predictive accuracy, but the models excelled either at screening or confirmation, not both. The addition of pNGAL and CRP significantly enhanced AKI prediction, emphasizing the need for biomarker integration in risk stratification models. These findings highlight the limitations of clinical parameters alone and underscore the importance of a multifaceted approach to AKI risk assessment.

## 1. Introduction

Acute kidney injury (AKI) is a global health issue, affecting millions of patients each year. It is associated with a high morbidity, mortality, and healthcare costs [1]. This global issue stems partly from the insufficient and/or delayed detection of AKI [2]. Until 2002, there were different definitions of AKI, making it difficult to compare studies. However, the introduction of the Risk, Injury, Failure, Loss of function, and End-stage kidney disease (RIFLE) staging system by the Acute Dialysis Quality Initiative (ADQI) [3] was followed by other standardized definitions of AKI, the Acute Kidney Injury Network (AKIN) [4] in 2005 and the international society of nephrology Kidney Disease: Improving Global Outcomes (KDIGO) [1] in 2012, aligning more recent studies. In 2012, KDIGO established specific risk factors contributing to non-specific acute kidney injury (AKI), categorizing them as exposures and susceptibilities. The KDIGO guideline recommends stratifying and managing patients at risk for AKI based on these factors, where the likelihood of AKI arises from the interaction between susceptibility factors and the type and degree of exposure to harmful insults. Susceptibility factors include dehydration, advanced age, female sex, black race, chronic kidney disease (CKD), diabetes mellitus, cancer, and anemia. Exposures encompass conditions such as sepsis, critical illness, circulatory shock, burns, trauma, cardiac and major non-cardiac surgeries, nephrotoxic drugs, radiocontrast agents, and toxic plants and animals [1].

The breadth and variability of these exposures and susceptibilities for AKI, as well as their potential for differing interpretations and definitions, pose significant challenges for cross-study comparisons and their practical application in clinical settings. Many studies have focused on identifying risk factors and interventions for AKI, but most conclude that while some strategies may reduce the incidence of AKI, the prevention of AKI remains a significant challenge, and more robust research is needed to identify effective interventions [5,6,7]. AKI’s etiology can be categorized into pre-renal, intrinsic, and post-renal types, each associated with distinct pathophysiological mechanisms, and different etiology types occur in various subpopulations. Hence, it is not surprising that assessing the risk of developing AKI is challenging [8]. Therefore, to minimize variability, scoring systems often target a specific disease or clinical scenario. AKI following cardiac surgery is likely the subpopulation with the most diverse and widely used scoring systems, focused on a specific clinical scenario [9]. Even though 10–20% of those admitted to the ED develop AKI, not many scoring systems have been developed for the emergency department (ED) setting. Phillips et al. developed a risk-stratification model for early ED-AKI. The model incorporated 8–10 variables readily available during ED triage, without relying on laboratory results. While the model was deemed acceptable and objective, the authors concluded that further refinement and external validation were necessary [10].

Beyond the challenge of AKI arising from various etiologies, developing a universal scoring system is further complicated by the widely variable prevalence of AKI among ED admissions, regional treatment practices, medical traditions, and patient prescreening protocols. Differences in AKI prevalence have a major impact on the performance of these scoring systems [11].

Furthermore, while KDIGO’s standardization of AKI diagnosis enhances consistency and reliability, the recommended biomarker, creatinine (Cr), has limitations, notably its delayed response to renal injury [12]. To address this, several alternative biomarkers have been proposed. Tissue metalloproteinase-2 (TIMP-2), insulin-like growth factor binding protein 7 (IGFBP7), C-reactive protein (CRP), and kidney injury molecule-1 (KIM-1) are among them [13,14]. Neutrophil gelatinase-associated lipocalin (NGAL), an acute-phase protein released by the kidneys in response to intrinsic AKI, is another frequently studied biomarker. However, because NGAL is also elevated during inflammation, this must be considered when interpreting its levels [14]. Our recent studies [15,16] have shown that plasma NGAL (pNGAL) is an excellent biomarker for ruling out AKI. To our knowledge, no studies have evaluated NGAL’s predictive performance for AKI in combination with KDIGO-defined exposures and susceptibilities.

Our objective was to assess the predictive significance of KDIGO-listed exposures and susceptibilities for AKI, utilizing KDIGO’s creatinine-based criteria within a Danish context, by analyzing data from our recent prospective longitudinal “NGAL study” [16]. We aimed to assess the predictive performance of KDIGO-defined exposures and susceptibilities for AKI [1], both as standalone factors and in combination with each other, within an acute and diverse ED population. Additionally, we wished to see if adding pNGAL and CRP to these risk factors could further improve their predictive properties.

## 2. Materials and Methods

### 2.1. Study Design and Setting

All data derive from our recent “NGAL study”, a prospective emergency department (ED) cohort study, where the study details are described [16].

The “NGAL study” was a prospective longitudinal study at North Zealand University Hospital, which is part of the Capital Region of Denmark and which conducts medical, surgical, level-2 trauma, emergency interventions, and intensive care around the clock. The ED at North Zealand Hospital is a general ED and treats approximately 50,000 patients annually [17,18]. For inclusion in the study, n three specific days each month, from 3 October 2019 to 26 December 2020, patients giving their consent and with an age of ≥18 years, admitted to the ED during the inclusion period, were included. Obstetric- and traumatically injured patients were excluded due to immediate admission to the obstetric department or orthopedic emergency room, respectively.

### 2.2. Data Collection

The data collected through medical records (Sundhedsplatformen (SP)) included medical diagnosis, age, gender, pharmaceutical medication, vital parameters, and laboratory test results. All data were merged using the Danish unique Central Personal Registry (CPR) number into the secure web-based Research Electronic Data Capture (REDCap)-database. The KDIGO guidelines [1] have identified various risk factors, categorized as exposures and susceptibilities, for non-specific AKI. These predefined risk factors, in the form of exposures and susceptibilities for non-specific AKI, were defined by the current study’s authors in detail (Table S1) before patient inclusion and systematically documented at the time of admission to assess their validity as predictors for AKI development. The Danish Society of Nephrology (DSN) [19] has included kidney transplantation as a susceptibility factor for AKI in their recommendations, and it is also considered in this study. Patients’ medical histories (medical evaluation and encounter data) were retrieved retrospectively, covering one year prior to inclusion, the admission period (including the entire hospitalization), and the following 12 months (up to a maximum of 10 hospitalizations post study inclusion). In addition to extracting information on KDIGO’s stated exposures and susceptibilities for AKI (as detailed in Table S1), investigators and medical students reviewed all medical records over the two-year period to document patients’ medical encounter data, comorbidities, and discharge diagnoses (Table S2).

### 2.3. AKI Definition

Patients were divided into AKI and non-AKI at admission. As the KDIGO [1] has different criteria for AKI, we chose to use an increase in pCr of ≥26.5 µmol/L from the prior twelve months’ mean baseline pCr value, as this made it possible to evaluate AKI in most of the patients in our previous study. All pCr values available for each patient during the twelve months were included [15].

### 2.4. Participants and Evaluating AKI Risk Assessment

**Figure 1:**
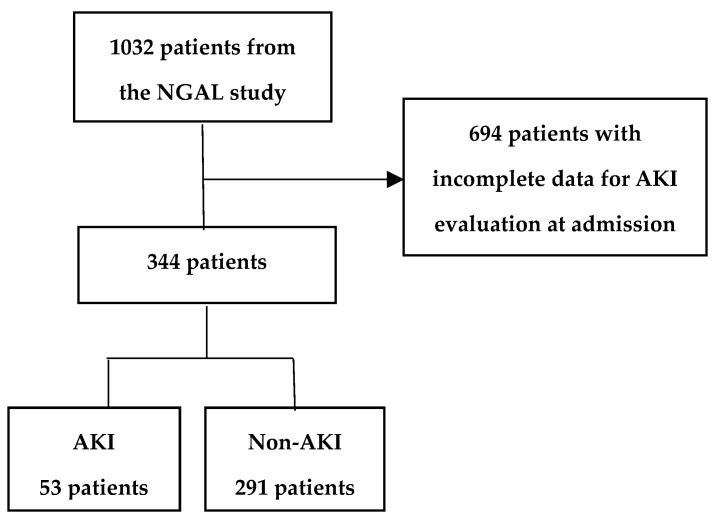
Flowchart of patient inclusion for study.

In this study, data on the risk factors described above, collected during admission and the corresponding hospitalization in the AKI cohort, were used to evaluate exposures and susceptibilities for AKI. As described in detail in the “NGAL study” [16], pNGAL levels measured at admission were also incorporated into the AKI assessment. Out of the 1032 patients included in the “NGAL study” [16], from 3 October 2019 to 26 December 2020, a total of 344 patients were eligible for AKI evaluation at admission and were included in the study cohort (Figure 1). Of these, 321 patients had both an AKI assessment and pNGAL measurements available at admission. There were not any restrictions regarding the value of pNGAL, thus all patients with a pNGAL measurement at admission were included.

A total of 344 patients out of the 1032 patients from the “NGAL study” [16] were included; 53 patients had AKI and 291 did not (non-AKI). AKI was defined by an increase in pCr of ≥26.5 µmol/L from the average baseline pCr value over the past twelve months. Non-AKI was defined by a change in pCr of <26.5 µmol/L from the average baseline pCr value over the past twelve months.

### 2.5. Statistical Method

Descriptive statistics are presented as median with IQR for continuous variables, as these were found to have a less skewed distribution, and frequencies and percentages for categorical variables.

The ability of the risk factors to predict AKI was analyzed using a receiver operating characteristic (ROC) analysis; this was accomplished through several models. First, we used separate ROC analyses for each risk factor, with AKI yes/no as the response and the specific risk factor as the only predictor, to evaluate the predictive capability of each risk factor. Secondly, every combination of the risk factor was included in separate ROC analyses. This was carried out by including the specific risk factors from a given combination as independent variables, varying from 2 to 21 variables, in a logistic regression model, with AKI yes/no as a dependent variable, again separately for each combination of risk factors. The predicted probability from the model was then used as a predictor in the ROC analysis, again with AKI yes/no as the response. An optimal cutoff of the predicted probability for each ROC analysis was determined from the maximum value of the sum of sensitivity and positive predictive value (SENSPPV), ranging from 0 to 2. We choose SENSPPV as an optimization measure, because we wanted to see if the risk factors could screen for AKI (sensitivity) and/or confirm AKI (PPV), preferably both. We did not make any selection of the variables or the number of variables included in the different logistic regression models used in the ROC analysis. This allowed for an evaluation of the optimal SENSPPV value for every possible combination of the risk factors, with 2,097,130 combinations in total. However, as we are not able to present results from the more than 2 million analyses, we have selected the combinations with the largest SENSPPV value. In cases with multiple combinations resulting in the same SENSPPV value, the combination with the fewest number of risk factors is presented, as this would suggest that the additional risk factors do not contribute to the SENSPPV value. We first present combinations not including pNGAL or CRP, with the 20 combinations with the largest SENSPPV value. Secondly, we present the top 10 combinations including pNGAL and the top 10 including CRP.

## 3. Results

### 3.1. Comparison of Medical Encounter, Diagnoses, and Conditions in AKI and Non-AKI Patients

The two-year period data, extracted from medical records at admission and one year pre and one year post admission, showed the number of occurrences for each diagnosis, procedure, or condition between AKI and non-AKI patients. Cardiovascular disease was the most common comorbidity (60% in AKI and 45% in non-AKI), followed by gastrointestinal (25% in AKI and 31% in non-AKI) and lung diseases (25% in AKI and 21% in non-AKI) in both patient groups. Kidney-related conditions (kidney insufficiency UNS (15%), pyelonephritis (8%), urosepsis (13%)) and infections (bacterial infections (8%), cystitis (28%), urosepsis (13%), sepsis UNS (19%)) were common among AKI patients. Anemia (13%) and urosepsis were slightly more frequent in AKI patients, with just one additional case respectively, compared to non-AKI patients. Further, only two kidney cysts and one kidney abscess were reported and all of these in AKI patients. Neither the one patient diagnosed with anuric kidney failure nor the two patients with hydronephrosis were diagnosed as AKI patients (Table S2).

### 3.2. Exposure and Susceptibility Distribution

The frequency varied between the exposure and susceptibility variables, with some having a very low frequency, such as burns with two (0.6%) cases and kidney transplantation with one (0.3%) case. In the non-AKI group, eight (2.4%) patients were identified as black; however, none of these patients were diagnosed with AKI (Table 1).

### 3.3. Exposures and Susceptibilities for AKI Assessment

A total of 14 of the exposure and susceptibility factors showed a poor individual performance in both sensitivity and PPV, with none having a SENSPPV value of more than 0.8, while kidney transplant and black race achieved a value of 1 in PPV and sensitivity. Respectively; the low frequency and skewed distribution in the sample is likely the explanation for this. Advanced age and nephrotoxic drugs had the best individual performance, with a sensitivity/PPV of 0.70/0.22 and 0.87/0.23, respectively (Table 2).

When using all exposure and susceptibility factors in combination models, SENSPPV values were increased, ranging from 1.231 to 1.255 among the top 20 best SENSPVV models (Table S3). The top 20 models had a similar performance within two types of clusters, either obtaining a PPV of 1 with a sensitivity around 0.24 (PPV cluster) or a sensitivity of 1 with a PPV around 0.24 (sensitivity cluster), with higher-sensitivity models requiring more variables (14–16) compared to high-PPV models, 10–13 variables (Table 3). When looking at the distribution of variables CKD, advance age and radioactive contrast were included in all the top 20 models; chronic heart disease, black race, cancer, diabetes mellitus (dm), burns, chronic liver, and chronic lung disease were in most of the models (14–18 models), with the remaining variables included in 9–11 models. These remaining variables appeared to be mostly distributed to either cluster, with kidney transplant, dehydration, and surgery in the PPV cluster and sepsis, critical illness/ICU, circulatory shock, nephrotoxic drugs, anemia, and sex in the sensitivity cluster (Table 3).

### 3.4. pNGAL and CRP for AKI Assessment

Using pNGAL alone for AKI assessment had the highest SENSPPV among the single variables of 1.3, with a sensitivity of 0.78 and PPV of 0.52. Using pNGAL in combination with exposures and susceptibilities factors further improved this to an SENSPPV value of 1.470, with a sensitivity of 0.79 and PPV of 0.68. Among the top 10 models, the SENSPPV did not drop much, with all at least 1.456, with small shifts in sensitivity and PPV. However, there were some changes in the number of variables included, with the third best model only including 12, compared to the best with 15 variables (Table 4).

CRP alone had an SENSPPV value of 1.1, with a sensitivity similar to pNGAL of 0.74 but a lower PPV of 0.37. In combination with the other variables, CRP had an improved SENSPPV of 1.441, again with a similar sensitivity of 0.82 and to a lesser extend a reduced PPV of 0.63, based on only 11 variables; however, all the best CRP models also included NGAL (Table 4). Restricting CRP models to not include NGAL reduced the SENSPPV to 1.341, with a sensitivity of 0.85 and PPV of 0.49 for the best model, but this model only consisted of eight variables (Table S4). Thus, the combination model integrating exposures, susceptibilities, and pNGAL outperforms both standalone clinical findings and biomarkers (Table 5).

## 4. Discussion

AKI risk arises from the interplay between patient susceptibility (e.g., age, CKD, comorbidities) and exposure to triggers (e.g., sepsis, surgery, nephrotoxic drugs). However, the relative impact of individual susceptibilities, exposures, and their combinations remains inadequately understood. Sepsis is the leading cause of AKI in critically ill patients, responsible for nearly 50% of cases, and it is associated with increased mortality [20,21]. Furthermore, both cardiac surgery [22] and contrast-induced AKI (CI-AKI) [23] are major causes of hospital-acquired AKI [23]. However, identifying true AKI risk factors remains challenging. Some patients may develop CI-AKI due to pre-existing conditions like CKD, diabetes mellitus, or advanced age, while others may be primarily at risk from the contrast agent itself. This complicates the determination of whether certain susceptibilities are general risk factors for AKI or specific to, in this case, contrast exposure.

In our current study, among the exposure and susceptibility variables, nephrotoxic drugs and advanced age showed a good sensitivity, but all exhibited a poor PPV (approximately 0.2). Likewise, dehydration, circulatory shock, chronic liver disease, and burns showed an okay PPV but with poor sensitivity. Black race and kidney transplantation stood out for having a sensitivity and PPV value of 1, respectively. However, the low frequency of these factors in the data likely accounts for these extreme values and may not accurately reflect performance in populations with higher frequencies.

The combination of multiple exposure and susceptibility variables to identify AKI overall improved the performance. However, the top 20 models could be separated into two clusters of models, with one having an optimal PPV at the cost of sensitivity and the other an optimal sensitivity at the cost of PPV. Sepsis, ICU, shock, nephrotoxic drugs, anemia, and sex presented almost exclusively in the sensitivity cluster, suggesting that the combined information contained in these variables are better at screening for AKI. Dehydration and surgery were mainly present in the PPV cluster, suggesting the combined information in these variables is better at confirming AKI. Contrast and advanced age were included in all 20 top models, suggesting that the information from these has an additive effect for both the screening and confirmation of AKI. This aligns with numerous studies that identify contrast as a risk factor for CI-AKI [23,24] and the many studies highlighting advanced age [25,26] as a significant risk factor for AKI.

Both pNGAL and CRP alone had some of the best predictive performance, with pNGAL alone having a better performance than the best exposures and susceptibilities combination model. The addition of pNGAL in the combination models resulted in an overall improvement, but all top models had similar values of sensitivity and PPV, rather than the distinct clusters for the exposure and susceptibility variable combinations. Similar to pNGAL, the addition of CRP in the combination models resulted in an overall improvement, with sensitivity and PPV values smaller than, but similar to, those for pNGAL. Even though the CRP models had a slightly worse performance than the pNGAL, the number of variables included was lower in the CRP models. However, all these models did also include pNGAL, and if models specifically excluding pNGAL were considered, performance was reduced but still better than the exposures and susceptibilities combination models. This could indicate that CRP contains more of the information contained in the exposures and susceptibilities variables than pNGAL and that pNGAL contains more additional information, based on the overall better performance.

A systematic review and meta-analysis [11] examined 150 studies on AKI prediction models, covering 14.4 million participants. It found that while these models generally have good discriminative power, their clinical utility is significantly limited due to a wide variability in study populations, methods, and outcome definitions. The heterogeneity in predictive variables, data collection approaches, and AKI definitions undermines model reliability and external validation. Despite many studies, no clear factors have been identified as primary drivers of this variability, and most models lacked external validation. The review highlights the need for standardized methods in model development, validation, and reporting to improve clinical applicability. The findings emphasize the urgency for more consistent methodologies to enhance the predictive value and real-world utility of AKI models [11].

Thus, in this current study none of the clinical parameters (exposures or susceptibilities) were particularly reliable, especially as a standalone, which may explain the challenges in detecting and preventing AKI. This highlights the need for biomarkers such as pNGAL and CRP, which demonstrated improved performance in AKI prediction in this study.

## 5. Conclusions

These combined insights emphasize the importance of a multifaceted approach to AKI risk assessment. In our analysis, no standalone exposures or susceptibilities were observed to have a good ability to identify AKI. Combinations of these variables led to improvements; however, each combination excelled at either screening for AKI or confirming AKI but not both. Both the addition of pNGAL and CRP improved the predictive performance, with pNGAL resulting in the highest performing combinations, but CRP did have a combination consisting of fever variables with a slight reduction in performance.

## Figures and Tables

**Table 1:** The frequency of exposures and susceptibilities for AKI between AKI and non-AKI patients.

Exposures and Susceptibilities ^^^ and pNGAL and CRP	AKI (n = 53)	Non-AKI (n = 291)	Total (n = 344)
Sepsis	11 (20.8%)	12 (4.1%)	23 (6.7%)
Critical illness/ICU	4 (7.5%)	8 (2.8%)	12 (3.5%)
Circulatory shock	4 (7.5%)	3 (1%)	7 (2%)
Burns	1 (1.9%)	1 (0.3%)	2 (0.6%)
Nephrotoxic drug	38 (71.7%)	127 (43.6%)	165 (48%)
Radioactive contrast	18 (34.6%)	64 (22.5%)	82 (24.3%)
Kidney transplant	1 (1.9%)	0 (0%)	1 (0.3%)
Diabetics mellitus	14 (26.9%)	49 (17%)	63 (18.5%)
Cancer	10 (18.9%)	34 (11.8%)	44 (12.9%)
Anemia	12 (22.6%)	17 (5.9%)	29 (8.5%)
Dehydration	15 (28.3%)	14 (4.8%)	29 (8.5%)
Surgery	10 (18.9%)	13 (4.5%)	23 (6.7%)
Chronic heart disease	10 (18.9%)	40 (13.7%)	50 (14.5%)
Chronic lung disease	16 (30.2%)	56 (19.2%)	72 (20.9%)
Chronic liver disease	5 (9.4%)	5 (1.7%)	10 (2.9%)
Advanced age	37 (69.8%)	135 (46.4%)	172 (50%)
CKD	17 (32.1%)	29 (10%)	46 (13.5%)
Black race	0 (0%)	8 (2.9%)	8 (2.4%)
Sex female	26 (49.1%)	166 (57%)	192 (55.8%)
pNGAL (median (IQR))	173.5 ng/mL (115.5:369.5)	59 ng/mL (50:83.5)	65 ng/mL (50:103)
CRP (median (IQR))	49 mg/L (12:108)	4 mg/L (3:13)	5.5 mg/L (3:23.75)
Creatinine (median (IQR))	127 µmol/L (92:212)	71 µmol/L (61:83.5)	73 µmol/L (63:90.5)

^^^ The definitions and interpretations of the various exposures and susceptibilities used in this study can be found in Table S1. Abbreviations: CKD = chronic kidney disease; CRP = C-reactive protein; ICU = intensive care unit; IQR = interquartile range; pNGAL = plasma neutrophil gelatinase-associated lipocalin.

**Table 2:** AKI assessment performance for each exposure and susceptibility.

Exposures and Susceptibilities and pNGAL and CRP	Sensitivity	Specificity	PPV	NPV	SENSPPV	Number of Patients ^^^	Number of Patients with AKI ^#^
pNGAL	0.78	0.87	0.52	0.96	1.30	321	50
Black race *	1.00	0.03	0.16	1.00	1.16	331	52
CRP	0.74	0.77	0.37	0.94	1.10	342	53
Kidney transplant	0.02	1.00	1.00	0.85	1.02	344	53
Nephrotoxic drug	0.72	0.56	0.23	0.92	0.95	344	53
Advanced age	0.70	0.54	0.22	0.91	0.91	344	53
Dehydration	0.28	0.95	0.52	0.88	0.80	342	53
CKD	0.32	0.90	0.37	0.88	0.69	342	53
Sex male	0.51	0.57	0.18	0.86	0.69	344	53
Sepsis	0.21	0.96	0.48	0.87	0.69	343	53
Circulatory shock	0.08	0.99	0.57	0.85	0.65	343	53
Anemia	0.23	0.94	0.41	0.87	0.64	343	53
Surgery	0.19	0.96	0.43	0.87	0.62	342	53
Chronic liver disease	0.09	0.98	0.50	0.86	0.59	344	53
Radioactive contrast	0.35	0.78	0.22	0.87	0.57	337	52
Chronic lung disease	0.30	0.81	0.22	0.86	0.52	344	53
Burns	0.02	1.00	0.50	0.85	0.52	343	53
Diabetics mellitus	0.27	0.83	0.22	0.86	0.49	341	52
Cancer	0.19	0.88	0.23	0.86	0.42	341	53
Critical illness/ICU	0.08	0.97	0.33	0.85	0.41	343	53
Chronic heart disease	0.19	0.86	0.20	0.85	0.39	344	53

* model predicts black race to be non-AKI; ^^^ the number of patients observed for the specific exposure, susceptibility, pNGAL, or CRP; ^#^ the number of patients with AKI in the observed patients. The table presents the performance of AKI assessment for each exposure and susceptibility factor, as well as the biomarkers NGAL and CRP. The performance measures single-variable models and was analyzed using logistic regression. Abbreviations: ICU = intensive care unit; NPV = negative predictive value; PPV = positive predictive value; SENSPPV = sum of sensitivity and PPV.

**Table 3:** Exposures’ and susceptibilities’ most effective predictive performance for AKI risk stratification in combination models.

The Most Effective Predictive Analysis	Exposures and Susceptibilities
High-PPV cluster	kidney transplant, dehydration, surgery
High-sensitivity cluster	sepsis, critical illness/ICU, circulatory shock, nephrotoxic drugs, anemia, sex
AKI risk assessment	radioactive contrast, advanced age, CKD

This table illustrates how different exposures and susceptibilities in different combination models enhance AKI prediction, aiding in risk stratification. The high-sensitivity cluster is useful for broad AKI screening, while the high-PPV cluster helps confirm AKI with greater specificity. Additionally, some exposures and susceptibilities contribute to AKI risk assessment, as they are present in all top models, reinforcing their universal contribution. These insights can guide clinicians in refining their approach to AKI screening and confirmation. The exact combination of the top 20 combination models can be found in Table S3. Abbreviation: AKI = acute kidney injury; CKD = chronic kidney disease; ICU = intensive care unit; PPV = positive predictive value

**Table 4:** Top 10 exposure–susceptibility combination models including pNGAL and CRP.

Included Exposures and Susceptibilities with pNGAL or CRP	Number of Variables Included	Sens.	Spec.	PPV	NPV	Sens-PPV
pNGAL	1	0.780	0.870	0.520	0.960	1.300
seps shock burn ND kdntrans dm canc anem surg chd lung liver black sex NGAL_first	15	0.792	0.930	0.679	0.960	1.470
seps shock burn ND kdntrans dm canc anem surg chd ckd black NGAL_first	13	0.854	0.898	0.612	0.970	1.466
icu shock burn ND kdntrans dm canc anem chd ckd black NGAL_first	12	0.833	0.907	0.625	0.967	1.458
seps icu shock burn ND kdntrans dm canc anem chd ckd black NGAL_first	13	0.833	0.907	0.625	0.967	1.458
seps shock burn ND kdntrans dm anem surg chd lung liver black sex NGAL_first	14	0.792	0.927	0.667	0.960	1.458
shock burn ND kdntrans dm canc anem surg chd lung liver black sex NGAL_first	14	0.792	0.926	0.667	0.960	1.458
seps shock burn ND kdntrans dm anem surg chd lung liver ckd black sex NGAL_first	15	0.792	0.926	0.667	0.960	1.458
seps shock burn ND kdntrans dm surg chd lung liver ckd black sex NGAL_first	14	0.771	0.934	0.685	0.956	1.456
seps shock burn ND kdntrans dm canc surg chd lung liver ckd black sex NGAL_first	15	0.771	0.934	0.685	0.956	1.456
seps shock burn ND kdntrans dm canc anem surg chd lung liver ckd black sex NGAL_first	16	0.771	0.934	0.685	0.956	1.456
CRP	1	0.740	0.770	0.370	0.940	1.100
seps icu shock ND canc anem liver age_65 black NGAL_first CRP_first	11	0.816	0.907	0.625	0.963	1.441
seps icu shock ND canc anem lung liver age_65 black NGAL_first CRP_first	12	0.816	0.907	0.625	0.963	1.441
seps icu shock ND canc anem liver age_65 ckd black NGAL_first CRP_first	12	0.816	0.907	0.625	0.963	1.441
seps icu shock ND canc anem lung liver age_65 ckd black NGAL_first CRP_first	13	0.816	0.907	0.625	0.963	1.441
seps icu shock ND canc anem lung liver age_65 black sex NGAL_first CRP_first	13	0.816	0.907	0.625	0.963	1.441
seps shock ND canc anem chd lung liver age_65 black NGAL_first CRP_first	12	0.796	0.915	0.639	0.959	1.435
seps shock ND anem chd lung liver age_65 black sex NGAL_first CRP_first	12	0.796	0.915	0.639	0.960	1.435
seps shock ND canc anem chd lung liver age_65 black sex NGAL_first CRP_first	13	0.796	0.915	0.639	0.959	1.435
seps icu shock burn ND canc anem liver age_65 black NGAL_first CRP_first	12	0.816	0.903	0.615	0.963	1.432
seps icu shock ND canc anem liver age_65 black sex NGAL_first CRP_first	12	0.816	0.903	0.615	0.963	1.432

The table presents the top 10 exposure–susceptibility combination models for AKI assessment, including pNGAL and CRP, evaluated using logistic regression. Abbreviation: age_65 = advanced age > 65 years; anem = anemia; black = black race; canc = cancer; chd = chronic cardiac disease; ckd = chronic kidney disease; CRP_first = first CRP at admission; contrast = radioactive contrast; dehy = dehydration; dm = diabetes mellitus; icu = intensive care unit = critical illness; kdtrans = kidney transplant; liver = chronic liver disease; lung = chronic lung disease; ND = nephrotoxic drug; NGAL_first = first NGAL at admission; NPV = negative predictive value; PPV = positive predictive value; Sens. = sensitivity; SENSPPV = sum of sensitivity; seps = sepsis; shock = circulatory shock; Spec. = specificity; surg = surgery.

**Table 5:** Performance of the top exposure–susceptibility combination model including and excluding pNGAL, pNGAL alone, and CRP alone for AKI assessment.

Variables	Sens.	Spec.	PPV	NPV	SENSPPV
seps shock burn ND kdntrans dm canc anem surg chd lung liver black sex NGAL_first	0.792	0.930	0.679	0.960	1.470
pNGAL	0.780	0.870	0.520	0.960	1.300
burn contrast kdntrans canc dehy surg chd lung liver age_65 ckd black	0.255	1.000	1.000	0.878	1.255
CRP	0.740	0.770	0.370	0.940	1.100

The table includes the best exposure–susceptibility combination model with and without pNGAL, pNGAL alone, and CRP alone, along with the performance in AKI assessment. Abbreviation: anem = anemia; black = black race; canc = cancer; chd = chronic cardiac disease; dehy = dehydration; dm = diabetes mellitus; kdtrans = kidney transplant; liver = chronic liver disease; lung = chronic lung disease; ND = nephrotoxic drug; NGAL_first = first NGAL at admission; NPV = negative predictive value; PPV = positive predictive value; Sens. = sensitivity; SENSPPV = sum of sensitivity; seps = sepsis; shock = circulatory shock; Spec. = specificity; surg = surgery.

## Data Availability

This study was approved by the Danish Data Protection Agency (HGH-2016-085, I-suite-nr: 02439). A pseudonymized version of data may be requested by contacting the corresponding author Wetterstrand V. (vickywetterstrand@gmail.com). An approval of the request from the Danish Data Protection Agency will, however, be needed before transfer of data can be carried out; the corresponding author will assist with this request.

## Supplementary Materials

The following supporting information can be downloaded at: https://www.mdpi.com/article/10.3390/jcm14082572/s1, Table S1: KDIGO’s defined causes of AKI: exposures and susceptibilities for non-specific AKI; Table S2: Medical evaluation and encounter data over a two-year period in AKI and non-AKI Patients; Table S3: The top 20 models of exposure- susceptibilities combination performances; Table S4: Performance measures exposures and susceptibilities combination models with CRP and excluding pNGAL. Top 10 models.

## Abbreviations

The following abbreviations are used in this manuscript:

AKI	acute kidney injury
AUC	area under the curve
b-pCr	baseline plasma creatinine
CI- AKI	Contrast-induced AKI
CKD	chronic kidney disease
CRP	C-reactive protein
dm	diabetes mellitus
ED	emergency department
eGFR	estimated glomerular filtration rate
GFR	glomerular filtration rate
ICU	intensive care unit
IQR	interquartile range
KDIGO	Kidney Disease: Improving Global Outcomes
mb-pCr	mean baseline plasma creatinine
ND	nephrotoxic drug
NGAL	neutrophil gelatinase-associated lipocalin
NPV	negative predictive value
pCr	plasma creatinine
pNGAL	plasma NGAL
PPV	positive predictive value
ROC	receiver operating characteristic
sCr	serum creatinine
UNS	unspecified
