# Supplementary material for: Unpacking KDIGO Guidelines: Prioritizing and Applying Exposures and Susceptibilities for AKI in Clinical Practice
Source: J Clin Med. 2025 Apr 9;14(8):2572. doi: 10.3390/jcm14082572 (PMC12027667; doi:10.3390/jcm14082572)
Supplement: Supplementary file 1 [file jcm-14-02572-s001.zip › jcm-3540973-supplementary.pdf]

**Table S1.** KDIGO´s defined causes of AKI: exposures and susceptibilities for non-specific AKI

| | |
|---|---|
| ***Exposures*** | |
| • **Sepsis**- suspected or proven infection (leucocytes >12 x $10^9$/l or <4 x $10^9$/l) + qSOFA >= 2 criteria (RF> 22/min. / changed behavior /confused /systolic BP <= 100mmHg | |
| • **Critical illness**- hospitalized in the intensive care unit (ICU) | |
| • **Circulatory shock**- hypotension (systolic BP <90mmHg or MAP <65mmHg) + hyperlactatemia (>= 2mmol/L) | |
| • **Burns** ≥ 2. degree (includes epidermis and dermis) | |
| • **Trauma** – excluded as these patients aren’t admitted to the study’s medical emergency department | |
| • **Radioactive contrast** - during hospitalization | |
| • **Nephrotoxic drugs**: aminoglycosides, Non-Steroidal Anti-Inflammatory Drug/NSAID, loop-diuretics, antihypertensive medicine (*angiotensin-converting-enzyme inhibitor* (*ACE-inhibitor)* or angiotensin II-receptor blocker (*ARB*) or spironolactone in hypovolemia patients | |
| • **Major surgery including cardiac surgery**= any invasive operative procedure in which a more extensive resection is performed, e.g. a body cavity is entered, organs are removed, normal anatomy is altered or if a mesenchymal barrier is opened (pleural cavity, peritoneum, meninges) | |
| ***Susceptibilities*** | |
| • **Chronic heart-/lunge/-liver disease:** | |
| **Heart** | |
| | Transplantation (heart-, lunge or liver) due to chronic organ dysfunction |
| | Chronic ischemic heart diseases |
| | Chronic heart failure: HFrEF (Heart failure with Reduced Ejection Fraction) with left ventricle EF ≤40% or HFpEF (Heart failure with Preserved Ejection Fraction) with clinical and objective heart failure signs but with normal left ventricle EF (>50%) |
| | Heart valve disease: Mitral insufficiency, Mitral stenosis, Aortic insufficiency, Aortic stenosis, Right- sided (tricuspid- and/or pulmonale-) valve insufficiency or stenosis, |
| | Heart valve replacement (biological or mechanic or catheter-based), MitraClip, Balloon dilatation/PCI of mitral valve. |
| **Lungs** | Chronic obstructive pulmonary disease (COPD) + receives medicine for this purpose, at least one time over the last year. |
| | Asthma and receives medicine for this purpose |
| | Chronic restrictive pulmonary disease/parenchymal lung disease and receives medicine for this purpose |
| **Liver** | Cirrhosis: alcoholic/ autoimmune/ chronic hepatitis B or C/ hemochromatosis/ non-alcoholic steatohepatitis/ secondary biliary cirrhosis (due to bile duct obstruction)/ medical hepatitis/ Alfa-1-antitrypsinmangel caused |

| | |
|---|---|
| | hepatitis/chronic hepatomegaly/ idiopathic/ infection- or venous occlusive disease caused<br><br>Chronic hepatitis B or C, bile- obstructive liver disease confirmed with a biopsy, autoimmune liver disease<br><br>Known for an alcohol over-consumption + increased liver-blood tests (ASAT/ALAT-ratio > 2,0) |
| • **Advanced age** > 65 years old | |
| • **Sex-Female gender** | |
| • **Black race** = either confirmed through picture in medical journal and/or described in medical journal | |
| • **CKD**= diagnose already stated in medical journal | |
| • **Kidney transplantation** | |
| • **Diabetes mellitus**= stated as known with diabetes mellitus or HbA1c ≥ 48 mmol/mol (6,5 %) and/or fasting plasma glucose ≥ 7.0 mmol/l or non-fasting plasma glucose ≥ 11.1 mmol/l | |
| • **Cancer**= all patients diagnosed with any type of cancer form, documented in medical journal. First excluded when relapse-free >= 5 years | |
| • **Persistent anemia**= female < 7 mmol/L and male <8 mmol/L for >= 3 months | |
| • **Dehydration**= received iv fluid during hospitalization + clinical journal-description of "dehydrated patient" | |

The table outlines how various exposures and susceptibilities were defined and interpreted for patients prior to inclusion in the study.

Abbreviation: ALAT= alanine aminotransferase; ASAT= aspartate aminotransferase; BT=blood pressure; iv=intravenous; MP= Mean Arterial Pressure; qSOFA=quick Sequential Organ Failure Assessment ; RF=Respiratory Frequency

**Table S2.** Medical evaluation and encounter data over a two-year period in AKI and non-AKI patients

| Medical Evaluation and Encounter Data | AKI | Non-AKI | Total |
|---|---|---|---|
| **Number of patients** | **53 (100%)** | **291 (100%)** | **344** |
| Cardiovascular disease | 32 (60%) | 132 (45%) | 164 |
| Examination* | 16 (30%) | 101 (35%) | 117 |
| Gastrointestinal disease | 13 ( 25%) | 90 (31%) | 103 |
| Lung disease | 13 (25%) | 62 (21%) | 75 |
| Unwell UNS | 8 (15%) | 67 (23%) | 75 |
| Procedure^ | 7 (13%) | 56 (19%) | 63 |
| Cystitis (bladder infection) | 15 (28%) | 41(14%) | 56 |
| Other infection (not bacterial) | 12 (23%) | 40 (14%) | 52 |
| Trauma | 8 (15%) | 37 (13%) | 45 |
| Dyspnea | 11 (21%) | 30 (10%) | 41 |
| None# | 4 (8%) | 35 (12%) | 39 |
| Cerebral catastrophe (stroke or severe brain event) | 0 (0%) | 36 (12%) | 36 |
| Bleeding | 9 (17%) | 23 (8%) | 32 |
| Cardiac arrhythmia | 2 (4%) | 25 (9%) | 27 |
| Surgery | 7 (13%) | 19 (7%) | 26 |
| Abscess, fistula, and hernia | 5 (9%) | 20 (7%) | 25 |
| Neurological disorder | 4 (8%) | 19 (7%) | 23 |
| Upper gastrointestinal disease | 3 (6%) | 19 (7%) | 22 |
| Constipation | 2 (4%) | 20 (7%) | 22 |
| Sepsis UNS | 10 (19%) | 12 (4%) | 22 |
| Headache | 4(8%) | 17 (6%) | 21 |
| Back pain | 3(6%) | 18 (6%) | 21 |
| Orthopedic disorder | 4 (8%) | 16 (5%) | 20 |
| Liver and pancreas disease | 6 (11%) | 14 (5%) | 20 |
| Kidney insufficiency UNS | 8 (15%) | 12 (4%) | 20 |
| Bacterial infection | 4 (8%) | 14 (5%) | 18 |
| Extremity pain | 1 (2%) | 15 (5%) | 16 |
| Bladder and urinary stones | 2 (4%) | 13 (4%) | 15 |
| Local inflammation | 0 (0%) | 14 (5%) | 14 |
| Psychiatric disorder | 3 (6%) | 10 (3%) | 13 |
| Electrolyte imbalance | 4 (8%) | 9 (3%) | 13 |
| Anemia | 7 (13%) | 6 (2%) | 13 |
| Urosepsis | 7 (13%) | 6 (2%) | 13 |
| Death | 6 (11%) | 7 (2%) | 13 |
| Joint pain | 3 (6%) | 8 (3%) | 11 |
| Medication side effect | 1 (2%) | 10 (3%) | 11 |
| Pyelonephritis | 4(8%) | 7 (2%) | 11 |
| AKI | 8 (15%) | 3 (1%) | 11 |
| Alcohol-related | 7 (13%) | 4 (1%) | 11 |

| | | | |
|---|---|---|---|
| Skin disorder | 1 (2%) | 9 (3%) | 10 |
| Syndrome | 2 (4%) | 7 (2%) | 9 |
| Left against medical advice | 1 (2%) | 8 (3%) | 9 |
| Cancer | 2 (4%) | 6 (2%) | 8 |
| Pregnancy-related symptoms | 0 (0%) | 6 (2%) | 6 |
| Orthopedic disorder | 2 (4%) | 4 (1%) | 6 |
| Genital disorder | 0 (0%) | 5 (2%) | 5 |
| Seizures UNS | 0 (0%) | 5 (2%) | 5 |
| Blood per rectum | 1 (2%) | 4 (1%) | 5 |
| Hematuria | 2 (4%) | 3 (1%) | 5 |
| Other endocrine diseases | 1 (2%) | 4 (1%) | 5 |
| Inflammatory joint disease | 1 (2%) | 3 (1%) | 4 |
| Hypoglycemia | 0 (0%) | 4 (1%) | 4 |
| Edema | 2 (4%) | 2 (1%) | 4 |
| Urinary retention | 1 (2%) | 3 (1%) | 4 |
| Medication abuse | 0 (0%) | 4 (1%) | 4 |
| Delirium | 3 (6%) | 0 (0%) | 3 |
| Diabetes | 1 (2%) | 2 (1%) | 3 |
| Tachycardia | 0 (0%) | 3 (1%) | 3 |
| Visual disturbance | 0 (0%) | 2 (1%) | 2 |
| Ascites (abdominal fluid) | 1 (2%) | 1 (0%) | 2 |
| Pneumothorax | 1 (2%) | 1(0%) | 2 |
| Hyperglycemia | 1 (2%) | 1 (0%) | 2 |
| Kidney cyst | 2 (4%) | 0 (0%) | 2 |
| Drug abuse | 0 (0%) | 2 (1%) | 2 |
| Hydronephrosis | 0 (0%) | 2 (1%) | 2 |
| Genital disorder | 0 (0%) | 2 (1%) | 2 |
| Adrenal insufficiency | 0 (0%) | 1 (0%) | 1 |
| Social reasons | 1 (2%) | 0 (0%) | 1 |
| Hallucinations | 1 (2%) | 0 (0%) | 1 |
| Septic shock | 1 (2%) | 0 (0%) | 1 |
| CKD | 0 (0%) | 1 (0%) | 1 |
| Nephrostomy dysfunction | 0 (0%) | 1 (0%) | 1 |
| Catheter dysfunction | 0 (0%) | 1 (0%) | 1 |
| Kidney abscess | 1 (2%) | 0 (0%) | 1 |
| Anuric kidney failure | 0 (0%) | 1 (0%) | 1 |
| SIADH (syndrome of inappropriate antidiuretic hormone secretion) | 0 (0%) | 1 (0%) | 1 |
| Hypocalcemia | 0 (0%) | 1 (0%) | 1 |

The table categorizes medical evaluations and encounter data for patients with AKI and non-AKI, showing the number of occurrences for each diagnosis, procedure, or condition over a period of two years.
*Examination= telemetry, EEG, ECG, objective body examination, CT-cerebrum, wound cleaning or blood sugar checkup ; ^procedure= endoscopy, skin incision, cardiac catheterization, percutaneous transhepatic cholangiography/bile duct drainage, direct current cardioversion, drainage, pleurocentesis, PEG tube placement or Extracorporeal Shock Wave Lithotripsy of ureteral stone;#None= not possible to interpret from medical records

**Table S3.** The top 20 models of exposure- susceptibilities combination performances

| Included exposures & susceptibilities | Number of variables included | Sens. | Spec. | PPV | NPV | SENSPPV |
|---|---|---|---|---|---|---|
| burn contrast kdntrans canc dehy surg chd lung liver age_65 ckd black | 12 | 0.255 | 1.000 | 1.000 | 0.878 | 1.255 |
| burn contrast kdntrans dm dehy surg chd lung liver age_65 ckd black | 12 | 0.240 | 1.000 | 1.000 | 0.878 | 1.240 |
| burn contrast kdntrans dehy surg lung liver age_65 ckd black | 10 | 0.235 | 1.000 | 1.000 | 0.875 | 1.235 |
| burn contrast kdntrans dm dehy surg chd lung liver age_65 ckd | 11 | 0.235 | 1.000 | 1.000 | 0.879 | 1.235 |
| burn contrast kdntrans canc dehy surg lung liver age_65 ckd black | 11 | 0.235 | 1.000 | 1.000 | 0.875 | 1.235 |
| burn contrast kdntrans dehy surg chd lung liver age_65 ckd black | 11 | 0.235 | 1.000 | 1.000 | 0.875 | 1.235 |
| contrast kdntrans canc dehy surg chd lung liver age_65 ckd black | 11 | 0.235 | 1.000 | 1.000 | 0.875 | 1.235 |
| burn contrast kdntrans dm canc dehy surg chd lung liver age_65 ckd | 12 | 0.235 | 1.000 | 1.000 | 0.878 | 1.235 |
| icu burn contrast kdntrans dm canc dehy surg chd lung liver age_65 ckd | 13 | 0.235 | 1.000 | 1.000 | 0.878 | 1.235 |
| seps icu shock burn any contrast dm canc anem dehy chd lung age_65 ckd black sex | 16 | 0.980 | 0.469 | 0.253 | 0.992 | 1.233 |
| seps icu shock ND contrast dm canc anem chd age_65 ckd black sex | 13 | 1.000 | 0.392 | 0.231 | 1.000 | 1.231 |
| seps icu shock burn ND contrast dm canc anem chd age_65 ckd black sex | 14 | 1.000 | 0.392 | 0.231 | 1.000 | 1.231 |
| seps icu shock ND contrast dm canc anem chd lung age_65 ckd black sex | 14 | 1.000 | 0.392 | 0.231 | 1.000 | 1.231 |
| seps icu shock ND contrast dm canc anem chd liver age_65 ckd black sex | 14 | 1.000 | 0.392 | 0.231 | 1.000 | 1.231 |
| shock burn ND contrast dm canc anem chd lung liver age_65 ckd black sex | 14 | 1.000 | 0.392 | 0.231 | 1.000 | 1.231 |
| seps icu shock burn ND contrast dm canc anem chd lung age_65 ckd black sex | 15 | 1.000 | 0.392 | 0.231 | 1.000 | 1.231 |
| seps icu shock burn ND contrast dm canc anem chd liver age_65 ckd black sex | 15 | 1.000 | 0.392 | 0.231 | 1.000 | 1.231 |
| seps icu shock ND contrast kdntrans dm canc anem chd liver age_65 ckd black sex | 15 | 1.000 | 0.392 | 0.231 | 1.000 | 1.231 |
| seps icu shock ND contrast dm canc anem chd lung liver age_65 ckd black sex | 15 | 1.000 | 0.392 | 0.231 | 1.000 | 1.231 |
| seps icu burn ND contrast dm canc anem chd lung liver age_65 ckd black sex | 15 | 1.000 | 0.392 | 0.231 | 1.000 | 1.231 |

The table showcases the top 20 exposure-susceptibility combination models for AKI assessment, evaluated using logistic regression, including the specific risk factors from a given combination as independent variables, varying from 2 to 21 variables, in a logistic regression model, with AKI yes/no as a dependent variable.

Abbreviation: age_65= advance age > 65 years; anem= anemia; black = black race; canc=cancer; ; chd=chronic cardiac disease; ckd= chronic kidney disease; contrast= radioactive contrast; dehy=dehydration; dm=diabetes mellitus; icu= intensive care unit= critical illness; kdtrans= kidney transplant; liver= chronic liver disease; lung= chronic lung disease, ND= nephrotoxic drugs; NPV= negative predictive value; PPV=positive predictive value; Sens.= sensitivity; SENSPPV=sum of sensitivity; seps= sepsis; shock= circulatory shock; Spec.=specificity; surg= surgery

**Table S4.** Performance* measures exposures and susceptibilities combination models CRP and excluding pNGAL Top 10 models

| Included exposures & susceptibilities | Number of variables included | Sensitivity | Specificity | PPV | NPV | SENSPPV |
|---|---|---|---|---|---|---|
| ND kdntrans anem surg liver black sex CRP_first | 8 | 0.846 | 0.837 | 0.494 | 0.967 | 1.341 |
| seps shock ND kdntrans anem surg liver age_65 black sex CRP_first | 11 | 0.827 | 0.851 | 0.512 | 0.963 | 1.339 |
| seps icu shock ND kdntrans anem surg liver age_65 black sex CRP_first | 12 | 0.827 | 0.851 | 0.512 | 0.963 | 1.339 |
| ND kdntrans anem surg liver black CRP_first | 7 | 0.846 | 0.833 | 0.489 | 0.966 | 1.335 |
| burn ND kdntrans anem surg liver black CRP_first | 8 | 0.846 | 0.833 | 0.489 | 0.966 | 1.335 |
| burn ND anem surg liver black sex CRP_first | 8 | 0.846 | 0.833 | 0.489 | 0.966 | 1.335 |
| icu ND kdntrans anem surg chd liver black CRP_first | 9 | 0.846 | 0.833 | 0.489 | 0.966 | 1.335 |
| icu ND kdntrans anem surg liver black sex CRP_first | 9 | 0.846 | 0.833 | 0.489 | 0.966 | 1.335 |
| shock ND kdntrans anem surg liver black sex CRP_first | 9 | 0.846 | 0.833 | 0.489 | 0.966 | 1.335 |
| icu burn ND kdntrans anem surg liver black sex CRP_first | 10 | 0.846 | 0.833 | 0.489 | 0.966 | 1.335 |

*Performance was evaluated using logistic regression model.

Abbreviation: age_65= advance age > 65 years; anem= anemia; black = black race; canc=cancer; ; chd=chronic cardiac disease; ckd= chronic kidney disease; CRP_first= first CRP at admission; contrast= radioactive contrast; dehy=dehydration; dm=diabetes; icu= intensive care unit; kdtrans= kidney transplant; liver= chronic liver disease, lung= chronic lung disease, ND= nephrotoxic drugs; NPV= negative predictive value; PPV=positive predictive value; SENSPPV=sum of sensitivity and PPV; surg= surgery
